# Prevention of depression and anxiety in later life: design of a randomized controlled trial for the clinical and economic evaluation of a life-review intervention

**DOI:** 10.1186/1471-2458-9-250

**Published:** 2009-07-20

**Authors:** Jojanneke Korte, Ernst T Bohlmeijer, Filip Smit

**Affiliations:** 1 University of Twente, Faculty of Behavioural Sciences, Psychology & Communication of Health & Risk, Citadel, Room H403 (JK) and H401 (ETB), P.O. Box 217, 7500 AE Enschede, the Netherlands; 2 EMGO Institute of Health and Care Research, VU University Medical Centre, Trimbos institute, Netherlands Institute of Mental Health and Addiction, P.O. Box 725, 3500 AS, Utrecht, the Netherlands

## Abstract

**Background:**

Depressive and anxiety symptoms in older adults could develop into significant health problems with detrimental effects on quality of life and a possibly poor prognosis. Therefore, there is a need for preventive interventions which are at once effective, acceptable and economic affordable.

**Methods and design:**

This paper describes the design of a study evaluating "The stories we live by", a preventive life-review group intervention, which was recently developed for adults of 55 years and over with depressive and anxiety symptoms. Both clinical and economic effectiveness will be evaluated in a pragmatic randomized controlled trial. The participants in the intervention condition will receive the 8-session preventive intervention. The participants in the control condition will have access to usual care. Clinical end-terms are depressive and anxiety symptoms, current major depressive episode, quality of life and positive mental health post-treatment (3 months after baseline) and at follow-ups (6 and 12 months after baseline). Additional goals of this study are to identify groups for whom the intervention is particularly effective and to identify the therapeutic pathways that are vital in inducing clinical change. This will be done by analyzing if treatment response is moderated by demographics, personality, past major depressive episodes, important life events and chronically disease, and mediated by reminiscence functions, perceived control, automatic positive thoughts and meaning in life. Finally the cost-effectiveness of the intervention relative to care as usual will be assessed by computing incremental costs per case of depression and anxiety avoided (cost-effectiveness) and per quality adjusted life year (QALY) (cost utility).

**Discussion:**

It is expected that both the life-review intervention and its evaluation will contribute to the existing body of knowledge in several ways. First, the intervention is unique in linking life-review with narrative therapy and in its focus on specific, positive memories. Second, the evaluation is likely to answer questions regarding the acceptability and cost-effectiveness of life-review that have not been addressed thoroughly until now. Positive results of this study will make available a new evidence-based intervention to improve public health among people of 55 years and over.

**Trial registration:**

Nederlands Trial Register TC = 1860.

## Background

Clinical depression and anxiety in older adults is a significant health problem carrying a poor prognosis [1-5], with prevalence rates reported as high as 8.8% to 23.6% [6,7] for depressive disorders and 1.2% to 15% for anxiety disorders [8].

The presence of depressive and anxiety symptoms which do not meet the diagnostic criteria are by far the most important risk factors of late-life depression and anxiety [9-11]. Therefore, there is a great need to develop effective, low-threshold preventive interventions for older people reducing the risk to develop clinical depression and anxiety. A recent meta-analysis shows that low-threshold, psychological interventions for older adults with depressive symptoms are indeed promising in preventing depressive disorders [12].

Several psychological interventions are currently available for reducing depressive and anxiety symptoms in older people, including psycho-educational approaches, cognitive behaviour therapy, psychodynamic therapy, and interpersonal therapy [13-17]. However, previous interventions are associated with low uptake rates. Hence, there is a need for interventions that are more acceptable. Life-review is currently gaining popularity, because this type of intervention can be specifically used in older adults. Life-review is characterized by a structured re-evaluation of one's own life, on the one hand aimed at coping with negative experiences and conflicts and on the other hand at giving a positive meaning to life [18-20]. Life-review's popularity could reflect the research findings that many -though not all- older adults tend to look back on and evaluate their lives [21], which seems to suggest that it will be acceptable for older people and might even be regarded as attractive. Moreover, research indicated that life-review leads to a strong reduction of depressive symptoms, comparable to the effects of cognitive behaviour therapy [22-26]. This method is probably also effective in reducing anxiety symptoms [27,28]. Jointly, this evidence appears to indicate that life-review is a promising venue to offering a possibly effective preventive intervention for older people otherwise not easily engaged in treatment for the milder manifestations of anxiety and depression.

With regard to reminiscing styles, Webster [29] differentiates among others between the functions of identity developing, problem solving, bitterness revival, and boredom reduction. The function "identity developing" uses memories actively in developing one's identity by discovering, clarifying and crystallizing important dimensions of the sense of who one is. "Problem solving", also known as instrumental reminiscence, refers to how memories of past coping strategies can be reused in the present. "Bitterness revival" is also about one's identity, but in a negative, complaining way. Instead of being integrated in the sense of who we are, negative experiences are constantly brought up. In "boredom reduction", the main goal is to escape from the present by romanticizing the past. Several studies indicate that reminiscence aimed at bitterness revival or boredom reduction is positively correlated with depression and anxiety, and negatively with wellbeing [30,31]. However, identity developing and problem solving are positively associated with psychological well-being [31] and successful aging [32].

The extent to which and why people reminiscence is also influenced by personality. Cully et al. [30] showed that neurotic behaviour is correlated positively with bitterness revival, boredom reduction and the overall level of reminiscence. This was borne out by Cappeliez and O'Rourke [33], who found that a higher score on neuroticism predicted higher scores on total reminiscence, identity and bitterness revival. More openness also predicted a higher total level of reminiscence (identity). The intra-psychical functions of reminiscence (boredom reduction, identity, bitterness revival) appear to be well predicted by personality characteristics.

Recently, life-review has become increasingly linked to other therapeutic theories aiming at people with clinically relevant mental distress [34]. For example, Watt and Cappeliez [25] developed a protocol in which life-review was linked to cognitive theories about depression. They proposed that life-review in depressive persons trigger recall of unrealistic, negative cognitions about themselves and life in general, and that these cognitions can be challenged and modified by both the therapist and clients. A promising combination of theories might be created by integrating life-review with narrative therapy. After all, a central assumption of narrative therapy is that life-stories are essentially a process of reconstruction of autobiographical memories [35,36]. Furthermore, narrative theory suggests that reminiscence in depressed older adults is likely to generate "problem-saturated" stories [37]. Characteristic of these stories is their (selective) focus on negative memories [38] often structured as a victimic plot [39], in which something good turns bad [40]. In contrast to cognitive theory, narrative therapy does not look for changing underlying cognitions on the basis of reality testing, but invites people to explore alternative stories (reconstructions) that may express optimism, a greater sense of mastery, and a fresh focus on positive experience. Different stories may unveil new meanings, and there is no "final truth" in those stories. At all times the individual client is the expert who determines which constructions of reality are meaningful [41].

We propose to integrate narrative therapy and life-review into a new and unified approach to alleviate depressive and anxiety symptoms in older people; "The stories we live by" [42]. The essence of the intervention is to discover stories about one's life that help the individual to lead a contented life. The memories people have are considered as both stories and social reconstructions of reality. It invites people to dwell on the meanings of their experiences and to evaluate those meanings. We expect that in this way, people can discover answers about what they regard as most important in their lives and which experiences were most valuable, in that they can give meaning to their lives [20]. Moreover, by looking back the coherence of the own life story increases. Bearger and McAdams [43] discovered a positive connection between the coherence of life stories and psychological well-being.

The literature offers different explanations for the efficacy of life-review in mental disorders, such as depression. However, until now there hardly has been any empirical evidence for the factors that moderate or mediate the effects of life-review on mental distress. A first explanation for the effectiveness of life-review is that depressive people have associations which have been reinforced by negative memories [44-46]. By consciously focusing on specific and positively charged memories, negative memories are likely to be pushed further to the background, while other positive memories will be retrieved more easily. Another explanation is that people experience a greater sense of control over their lives when looking back [19]. People become aware that they are much stronger than expected.

"The stories we live by" intervention was evaluated in a pilot study [34]. The results were promising and to make this life-review intervention available as a possibly evidence-based intervention for older people, we think a logical next step is to carry out a randomized controlled trial (RCT). This paper describes the design of a RCT to assess clinical and economic effectiveness. The understanding of moderating and mediating effects is an important focus of this study. Conceptually, analysis of moderators would help to indentify subgroups for whom the intervention is particularly (cost-)effective, whereas analysis of mediators may shed light on how treatment effects are relayed over various clinical pathways and have a final impact on clinical endpoints [47]. Therefore, the specific hypotheses that the RCT addresses are described below.

Our main hypothesis is that the life-review intervention leads to a significant reduction of depressive and anxiety symptoms, and in current major depression, and a significant improvement in quality of life and positive mental health, compared to the no-treatment control condition.

In addition, we explore if gender, age, education level, personality, past major depressive episodes, important life events and chronic diseases, in combination with the intervention, predict higher or lower effects on depressive and anxiety symptoms, quality of life and positive mental health.

Furthermore, we hypothesize that reminiscence functions, perceived control, automatic positive thoughts and meaning in life mediate the intervention's effects on clinical endpoints.

Finally, we expect that the incremental costs per case of depression and anxiety avoided (cost-effectiveness) and per quality adjusted life year (QALY; cost-utility) are lower in the intervention condition, compared to the care-as-usual condition.

## Methods and design

To evaluate the effectiveness of the life-review intervention, we will carry out a pragmatic RCT and a cost-effectiveness analysis (CEA). Participants will be randomly assigned to either the 8-session preventive intervention or the control condition; the care-as-usual condition. The research has been approved by the METiGG, a medical-ethics committee for research in mental health care settings in the Netherlands.

### Participants

An open recruitment strategy will be used, in cooperation with Dutch regional mental health care institutions, via advertisements in regional and national newspapers, information booklets available at health care institutions and general practitioners, plus a radio interview and commercial. Health care professionals at these mental health care centres (therapists, psychologists, etc.) will support the participant recruitment and intake, and eventually take care of organizing and offering the course at their centre. When people wish to participate, they will be invited by the nearest mental health care centre for an interview. In this interview, information is given about the intervention and the accompanying study, and the in- and exclusion criteria are examined.

The inclusion criteria are an age of 55 years or over, and the presence of slight to moderate depressive and anxiety symptoms. The presence of these symptoms will be measured by a score of 10 and above on the Dutch version of the Center for Epidemiological Studies Depression Scale (CES-D) [48] and a score of 3 and above on the Dutch version of the anxiety scale of the Hospital and Anxiety Scale (HADS-A) [49]. The CES-D is a 20-item self-report scale developed to measure depressive symptoms in the community [48]. Participants will be asked to indicate how often they experienced each symptom during the previous week. Response categories, ranging from 0 to 3, are "rarely or never", "some of the time", "occasionally", or "mostly or always". Summation results in a CES-D score, ranging from 0 to 60. A score of 16 or higher is considered indicative of clinically relevant depressive syndromes. The psychometric properties of the scale are found to be reliable in older populations [50], and more particularly in a sample of older Dutch people with depressive symptoms [51]. The HADS-A is a 7-item, self-report screening scale against which respondents are asked to indicate whether they had experienced feelings of restlessness, tenseness, or panic during the past four weeks [49]. Items range from 0 "rarely or never" to 3 "always or most of the time". The Dutch translation has shown good psychometric properties in six different groups of Dutch subjects [52]. Bjelland et al. (2002) [53] showed that among the general population and in somatic patients samples an optimal balance between sensitivity and specificity was achieved when caseness was defined by a score of 8 or above.

People will be excluded if diagnosed with full-blown depression or having a moderate to high suicide risk according the Dutch version of the Mini International Neuropsychiatric Interview (MINI). [54,55], a short diagnostic psychiatric interview to assess DSM-IV and ICD-10 disorders. The MINI was validated in a cross-national study involving more than 600 subjects. The concordance and psychometric values of the MINI Core, as compared to the CIDI [56] and the SCID-P [57], were found to be satisfactory. Furthermore, applicants scoring below the inclusion criteria of depressive and anxiety symptoms will be excluded, measured by a score of 9 and below on the Dutch version of the CES-D [48] and a score of 2 and below on the Dutch version of the HADS-A [49]. In addition, when applicants started taking anti-depressant medication or benzodiazepines recently (within the previous 2 months) or are currently receiving any psychological treatment they will be excluded. Finally, people will be excluded if the health care professionals assess that other serious psychopathology is present; then they are referred for psychological treatment. Applicants eligible to participate will be asked to sign an informed consent form.

### Randomization

Participants will be randomly assigned to either the experimental or control condition by means of a centrally conducted randomization process, stratified for gender and symptom level (no major depressive episode (0–4 symptoms) or slight (5 symptoms) to moderate (6–7 symptoms) major depressive episode). The randomization will be conducted at the University of Twente, independent of the participating mental health care centres. The mental health care centres and participants receive the outcome of the randomization by mail.

### Experimental condition

In total, 14 Dutch mental health care centres -in both rural and urban areas- have expressed their agreement to participate in this study. The therapists and prevention workers (from now on called therapists) at these centres -that will implement "The stories we live by"- all have therapeutic backgrounds or an education in behavioural sciences (e.g., psychology) or social work. For the purpose of offering the intervention they have received a two-day training program, which was supervised by a psychotherapist specialized in narrative therapy and a psychologist specialized in life-review. During the intervention, they will participate in two half-day follow-up meetings and a booster training.

"The three main components in "The stories we live by" are described below.

The first component is a focus on developing alternative, more positive, "thicker" life stories. Since the intervention is aimed at older adults with depressive and anxiety symptoms, we expect their stories to contain themes such as incapacity, disappointment and "being a victim". The therapists ask questions via deconstruction and reconstruction, two processes that are central in narrative therapy [58,59,37]. In the deconstruction phase, the therapists will explore values and norms that are inherent to the "problem-saturated stories" -and that may be inauthentic to the participant-, unique memories that seem to contradict the dominating problem-orientated story. An example of a deconstruction question that the therapist could ask is "Where there any exceptions (e.g. pleasant moments) in this difficult time of your life?". In the reconstruction phase, alternative, more authentic stories based on the participant's strength are constructed and "thickened". Examples of these questions are "How were you able to cope with this situation?", and "Now, at a much later date, can you say that you have also learned from that period in your life, could you explain?".

The second important component of the intervention is the systematic evaluation of one's life course, with a special focus on integrating negative life events with positive life events within participants' life stories. The intervention places the problems and conflicts that participants experience in the context of their life course, which involves making explicit links between the past and the present. This makes the intervention different from narrative therapy, where there is often much more focus on the present or themes the client feels like introducing into a session. During the intervention meetings, participants have to review their lives. Each intervention meeting has a different life theme, namely "our origin", "youth", "work and care", "love and conflicts", "loss and difficult periods", "metaphor", "the course of life" and "the future". Before each meeting, participants have to answer questions about different life themes. At home, they have to reflect upon these questions and write the responses down briefly. During the meetings, they have the opportunity to exchange and discuss the answers and experiences with the other participants. At home, participants can do creative assignments, which stimulate the imagination and may open up new ways of expression besides the verbal mode. To minimize resistance, we consciously choose to offer these assignments in a facultative manner. In the final meetings, attention is focused on the near future, to invite participants to convert their "new identity" into concrete actions.

The third important component of the intervention is the attention for specific positive memories, which are special and unique for a certain period in the participant's life. Participants have to write down exactly what they remember of this situation and describe it by means of the following questions: "Where was it?", "What did the environment look like?", "Which people where there and what did everyone look like?", "What happened exactly?". We expect that especially in depressive and anxious people these questions will activate memories that were "forgotten". This probably leads to a better balance between positive and negative memories, in that positive details are coming to the forefront again.

The intervention is aimed at four to six people and consists of eight similarly structured two-hour sessions. At the start of the sessions people are asked to explain into two or three sentences what they have learnt from the previous session with regard to their lives. Then, participants have to review their lives and exchange and discuss their experiences with the other participants. Finally, in each session attention is given to specific positive memories that the participants come up with.

### Control condition

Participants in the control condition receive no intervention. However, they have unrestricted access to care-as-usual and may receive all health care they desire. Moreover, participants are not withheld from any treatment (e.g. they may receive psychological treatment). In the context of the economic evaluation, health care uptake will be closely monitored. After conclusion of the RCT, the participants in the control condition will be invited to take part in the intervention.

### Measurements

#### Overview

Table 1 gives an overview of all measurements. Participants will be asked to complete questionnaires at baseline (t0), directly after the end of the intervention (t1), 3 months after the end of the intervention (t2), and -only in the intervention condition- 6 months after the end of the intervention (t3). The primary and secondary outcome measures will be recorded at all measurements (except for current major depressive episode), moderators only once at t0, the mediators at t0, t1 and t2 and the measures for economic evaluation only at t0 and t2.

**Table 1:** Measurement overview (intake, t0, t1, t2 and t3)

*Outcome measure*	*Instrument*	*t0*	*t1*	*t2*	t3*
*Primary outcome measure*					
Depressive symptoms	CES-D	X	X	X	X
					
*Secondary outcome measures*					
Current major depressive episode	MINI	X		X	
Anxiety symptoms	HADS-A	X	X	X	X
Quality of life	EQ 5-D	X	X	X	X
Positive mental health	MHC-SF	X	X	X	X
					
*Moderating measures*					
Demographics (age, gender and education level)		X			
Personality	NEO-FFI	X			
Past major depressive episodes	MINI	X			
Important life events		X			
Chronic diseases		X			
					
*Mediating measures*					
Reminiscence functions	RFS	X	X	X	
Perceived control	Mastery scale	X	X	X	
Automatic positive thoughts	ATQ-P	X	X	X	
Meaning in life	MLQ-SF	X	X	X	
					
*Measures for the economic evaluation*					
Resource use	TIC-P	X		X	
Production losses	PRODISQ	X		X	

*Only in intervention condition

#### Primary outcome measure

1. *Depressive symptoms *will be measured using the Dutch version of the CES-D [48].

#### Secondary outcome measures

2. The presence of *anxiety symptoms *will be assessed using the Dutch version of the HADS-A [49].

3. The presence of a *current major depressive episode *will be measured using the Dutch version of the MINI [54,55].

4. *Quality of life *will be determined using the Dutch version of the EuroQol (EQ-5D). The EQ-5D is a validated instrument for measuring health-related quality of life [60]. It covers five domains of health: mobility, self-care, usual activity, pain/discomfort and depression/anxiety. Each of the five domains has three severity levels; 0 (none), 1 (some) and 2 (severe). Permutation of the EQ-5D scores generates a total of 243 distinct health states, each of which is associated with a utility score ranging from 0 (poor health, similar to death) to 1 (perfect health). These utilities (also known as "tariffs") have been obtained in the UK and in the Netherlands [61,62]. In our study, we will use the Dutch EQ-5D tariffs to compute quality adjusted life years (QALYs) gained, and these will be used as outcome for the cost-utility analysis.

5. *Positive mental health *will be assessed using the Dutch version of the Mental Health Continuum Short Form (MHC-SF) [63]. The MHC-SF measures the mental health that according to Keyes is not simply the absence of mental illness, but the presence of positive feelings (emotional well-being) together with positive functioning in both individual life (psychological well-being) and community life (social well-being) [64]. The MHC-SF is a 14-item questionnaire and measures three dimensions: hedonic well-being (3 items), social well-being (5 items) and psychological well-being (6 items). Participants are asked how often they have experienced the feelings described in the items during the past month, using a 6-point answer format ranging from "never", "once or twice", "approximately once a week", "two or three times a day", "almost every day" to "every day". The MHC-SF has been shown to have good psychometric properties [65], also for the Dutch population [66].

#### Moderating measures

1. The *demographic*s that we will measure are age, gender and level of education.

2. Personality will be assessed using the Dutch version of the NEO-FFI [67,68]. This self-report questionnaire consists of 60 statements covering 5 main dimensions of personality: neuroticism, extraversion, openness to new experiences, agreeableness, and conscientiousness. Each statement is rated on a 5-point scale ranging from "strongly disagree" to "strongly agree," resulting in total dimension score between 12 and 60. The NEO-FFI has good psychometric properties [67,69].

3. Past major depressive episodes will be measured using the Dutch version of the MINI [54,55].

4. We will ask participants if they have any *chronic disorders*, such as coronary diseases, lung diseases, or rheumatism.

5. Participants have to report if any *critical life events *had occurred within in the previous three years, such as the lost of a spouse or a divorce.

#### Mediating measures

1. *Reminiscence functions *will be measured using the Dutch version of the Reminiscence Functions Scale (RFS), a valid and reliable 43-item questionnaire that assesses reminiscence functions over one's life course [70,71]. The scale comprises eight subscales (factors) reflecting possible functions of reminiscence for the individual. The subscales are labelled "boredom reduction", "death preparation", "identity", "problem-solving", "conversation", "intimacy maintenance", "bitterness revival", and "teach/inform". Questions typically start with "when I reminiscence, it is..." and are completed using 43 possible reasons or motivations to reminiscence. Respondents will be asked to indicate the extent to which each of the 43 reasons applies to them. Possible answers range from 1 to 6 (never, rarely, seldom, occasionally, often, or very often). For the purpose of this research, 23 questions covering four subscales have been selected: 6 about boredom reduction, 6 about identity, 6 about problem solving, and 5 about bitterness revival. Examples are: "When I reminisce, it is... to pass the time during idle or restless hours" (boredom reduction), "to see how my past fits in with my journey through life" (identity), "to help me plan for the future" (problem solving), or "to keep painful memories alive" (bitterness revival). Scores are averaged per subscale each, representing a reminiscence function. The higher the score, the more the indicated function prevails. In addition, the summary score of all items indicates the extent to which a person reminiscences, irrespective of the reason or function of reminiscence.

2. To assess the degree of *perceived control *over one's life, we will use the Dutch version of the Mastery Scale [72]. This consists of seven items that are intended to assess beliefs about perceived control over one's life in general or beliefs regarding one's ability to control an event. We use the abbreviated version of five items which are each phrased in a negative way. Possible responses are given on a 5-point scale ranging from 1 "strongly disagree" to 5 "strongly agree". Summary scores range from 5 to 25. Higher scores on the scale indicate lower levels of perceived control. The Mastery-Scale has good psychometric properties [72].

3. *Automatic positive thoughts *will be measured using the Dutch version of the Automatic Thoughts Questionnaire Positive (ATQ-P), which asks respondents to rate how frequently each of the 30 positive self-statements or a similar thought occurred during the preceding week on 5-point scales ranging from 1 (never) to 5 (all the time). The scale's total score represents the degree of overall positive thinking [73]. The ATQ-P has good psychometric properties in 257 Dutch bereaved adults [74].

4. *Meaning in life *will be measured using the Meaning in Life Questionnaire (MLQ), a 10-item measure of the presence of, and the search for, meaning in life [75]. We will use a Dutch version in which participants have to answer on a 5-point scale from 1 "absolutely untrue" to 5 "absolutely true" what they think makes their life feel important to them. The MLQ has good psychometric properties [75].

### Measures for the economic evaluation

The economic evaluation will be carried out using the Trimbos and Institute of Medical Technology Assessment Questionnaire on Costs Associated with Psychiatric Illness (TIC-P), the PROductivity and DISease Questionnaire (PRODISQ), and the EQ-5D. The costs of receiving formal or informal health care will be measured using the TIC-P [76]. Costs will be assessed from a societal perspective and include both direct and indirect costs related to receiving formal and informal health care. Production losses due to illness in the four weeks preceding the research, in terms of both paid and unpaid work, are covered by the relevant parts of the PRODISQ [77]. These costs arise when people are absent from work, or work less efficiently due to depression, for example. A lost working day will be monetarily valued using the gender and age specific value of productivity losses, as reported by Oostenbrink et al. [78] indexed for the reference year 2007. The EQ-5D health state valuations correspond with QALYs gained or lost when each person stays in a particular health state for precisely one year. For the health state valuations we will make use of the Dutch tariffs [62].

### Effectiveness analysis of primary and secondary outcome measures

To test the null-hypothesis of the intervention's non-inferiority relative to care-as-usual, analysis will be conducted on an intention-to-treat basis following the CONSORT guidelines [79]. To that end, missing observations at follow-up will be imputed. If there are missing values at different measurements due to drop-out, multiple imputation (MI) is used to replace the missing values with ten new estimates. Relative improvements in the clinical endpoints in the experimental condition as compared to the care-as-usual control condition will be evaluated with help of linear modelling (e.g., regression analysis for Gaussian distributed dependent variables). In linear modelling framework, adjustments for confounders can be carried out, when so required. In the likely absence of confounders, this approach is equivalent to conducting a t-test of the clinical endpoint on the conditions (coded 1 for the experimental and 0 the control condition). When the clinical endpoint of interest is not normally distributed, the test and the 95% confidence intervals will be based on robust standard errors (i.e., while using the first-order Taylor-series linearization method) or on non-parametric bootstrap techniques (with 2,500 bootstrap replications). This would help to ascertain the effectiveness of the treatment relative to the comparator condition.

### Power calculation and sample size

To demonstrate presence of an effect of at least 0.35 standard units, (considered to be the lower-bound of a medium-sized effect [80]) as statistically significant in a one-tailed test at alpha = 0.05 and a power of (1-beta) = 0.80 a minimum of 80 participants in each condition will be required at follow-up (power calculation in Stata 7.0). Anticipating a drop-out rate of 20% between t0 and t2, 100 participants per condition need to be included at t0. We will use one-tailed tests in our study, because our hypotheses are directional. Moreover, a one-tailed test provides more power to detect an effect and requires a smaller sample size, which is preferred from a medical-ethics and financial point of view.

### Analysis of moderators

Following Kraemer et al., moderating factors measured at baseline (t0) will be identified using multivariate regression analysis of the imputed clinical endpoints (at last follow-up) on the interaction terms of the putative moderators with the treatment dummy (alongside their constituent main-effects) [47]. In this way, it can be tested if some baseline characteristics of the participants are prognostically favourable (or not) when this particular intervention is offered. This knowledge may alert us to possible improvements of the intervention, and should that prove difficult, then to at least be able to improve referral of patients to this intervention (i.e., indications and contra-indications for a more optimal patient – treatment match).

### Analysis of mediating measures

Mediating analyses will be performed in accordance with the steps outlined by Kraemer et al. [47]. In step 1, the putative mediator (e.g. mastery) will be the dependent measure and the treatment the independent variable. This establishes that the intervention is correlated with the putative mediator. In step 2, the dependent measure will be the outcome measure (e.g. CES-D and HADS-A). The independent measures are: treatment (coded 1 for the experimental and -1 for the control condition, putative mediator (centred at the average of both conditions), and the "treatment × putative mediator" interaction. This will be done to assess whether there is either a main effect of the putative mediator or an interactive effect with treatment. In this model, the intercept is the mean response to both conditions. The main effect of treatment equals the treatment-control difference (i.e., the main effect of the mediator is the average slope of treatment and control). Finally, the interactive effect is the difference between the two slopes.

### Economic evaluation

The aim of the economic evaluation is to estimate the incremental cost-utility in terms of quality-adjusted life years (QALYs) and the cost-effectiveness in terms of avoided cases of depression and anxiety in the treatment condition as compared to the comparator condition. In addition, costs of the intervention itself will be estimated. Cost calculations will be conducted following the Netherlands guidelines for economic evaluations [78], and will be carried out from the societal perspective and with the time-horizon set at one year. Due to this short time-horizon, costs will not be discounted.

Production losses will be valued monetarily with help of the so called "friction cost" method. Costs and QALYs will be assessed while employing the intention-to-treatment method. If clinical and economic end-points are missing at follow-up, they will be replaced by expected values (based on MI). In addition, the incremental cost-effectiveness ratio (ICER) will be calculated, the ratio of the difference in costs (cost of intervention condition minus cost of comparator condition) divided by the difference in effects (effect of intervention condition minus effect of comparator condition) [81]. Its uncertainty will be graphically represented in the ICER plot with help of the bootstrap method (with 2,500 bootstrap replications), a statistical method based on repeatedly sampling from the observed data generated in an evaluation [82].

Finally, cost-effectiveness acceptability (CEAcc) curves will be plotted. CEAcc curves are a graphical representation of the probability that a particular intervention is cost-effective, over a range of possible values for the maximum willingness to pay for a unit improvement in health outcomes, λ [83]. However, given that the value of λ is unknown, the probability that the new intervention is relatively more cost-effective than existing practice is presented for a range of levels of willingness to pay. Thus a CEAcc curve is created by varying the value of l from zero to infinity [81]. In our study we ascertain how the likelihood of the intervention being more acceptable from a health economic point of view depends on the willingness to pay for a QALY. Sensitivity analyses will be carried out to ascertain the robustness of the findings under different scenarios, e.g. under varying values of the main cost drivers that represent the most significant expenses.

## Discussion

The purpose of this study is to evaluate the effectiveness of "The stories we live by" intervention, a recently developed life-review intervention. We predict that the experimental condition will show superior effects in reducing depressive and anxiety symptoms and in improving quality of life and positive mental health, and will be cost effective. Both the intervention itself and its evaluation are likely to add to the existing body of knowledge in several ways, as described below.

### Strengths and limitations of the life-review intervention

The life-review intervention is unique in linking life-review with narrative therapy and in its focus on specific positive memories. An advantage of life-review as an intervention method is that it connects with two activities often seen in older people: reminiscing and story-telling. Also, the method seems to suit people who struggle with identity and meaning-in-life questions, especially when people are confronted with traumatic or important life-events or with significant life transitions, which possibly could lead to confusion, and feelings of hopelessness. Life-review interventions may help people to come to stories of their lives and find starting points in their own experiences that may help to find their own identity.

However, the use of life-review as a method for therapeutic intervention may have limitations. A possible limitation is that participants' bitterness revival and feelings of victimization may be reinforced. This may happen when they over and over discuss their negatively coloured story. At the same time they are not able to distance themselves from it. When this is the case, the depressive symptoms exhibited by participants tend to increase, rather than decline. Life-review should not to be seen as a harmless method without the risk of inducing negative effects. Therefore, it is a priority that the life-review process is guided by experienced health care professionals. To ensure this, the therapists participating in our study our obliged to have a therapeutic background, or an education in behavioural sciences (e.g., psychology) or social work and have received a specific training program before working with participants. After this training, they are able to invite the participants -through directed questions- to come to new life stories and to continuously link the present with the near future.

### Strengths and limitations of the RCT

Our study will answer questions regarding the acceptability and effectiveness of life-review that have not been addressed until now. We will investigate who benefits most and will try to understand the clinical pathways that are responsible for inducing the treatment effect. For example, we have included the role of personality as a new potential moderator and we have included a measurement scale based on reminiscence styles -the Reminiscence Function Scale- to investigate if it functions as a mediator. In addition, we have added an economic evaluation, and are one of the first to do this regarding life-review (also in the study protocol by Pot et al. [84]). However, it should be noted that these additional analyses cannot be conducted in absence of a treatment effect.

Furthermore, our RCT gives us the opportunity to make generalizations, as the intervention will be studied in its natural setting and the recruitment strategy of the study is very similar to the actual recruitment of the mental health care centres that will offer "The stories we live by". Another strength of this RCT is the use the MINI, a structured diagnostic interview for DSM-IV and ICD-10 psychiatric disorders. This allows us to stratify the randomization by symptom level (no major depressive episode or slight to moderate major depressive episode) and to detect any changes in the status of major depressive episode.

We recognize some limitations in this study. First, the time available to study a change in the outcome measures was only six months. For ethical reasons, the participants in the control condition received the intervention 3 months after t1. For future research an extended period is advised. Second, there is no control condition at six-month follow-up after the course. Therefore, definite conclusions that the possible effects at six-month follow-up may be related to the intervention cannot be made. Third, the extended follow-up period was only nine months following the conclusion of the course, but longer follow-up periods are needed to investigate for how long the possible effects will persist.

Notwithstanding the limitations, the development and research of interventions that prevent depressive and anxiety symptoms from developing into full-blown depression and anxiety is of the utmost importance, as these severe and persistent mental disorders are associated with a large burden of disease and extensive economic costs. Due to the pragmatic nature of the RCT, results will show whether or not potential effects will hold in mental health care practice and can be considered ecologically valid. Presently, Dutch mental health care centres are highly interested in working with life-review interventions. Moreover, "The stories we live by" has the potential to be implemented in several target groups, as the intervention is for example already offered to Turkish and Moroccan older people. To conclude, when the hypothesis of the interventions superiority over care-as-usual can be demonstrated, there will be available a new evidence-based life-review intervention to alleviate depressive and anxiety symptoms among people of 55 years and over.

## Competing interests

The authors declare that they have no competing interests.

## Authors' contributions

All authors contributed to the design of the study. JK drafted the manuscript and will take care of the recruitment of participants and data collection. ETB helped to draft this manuscript. JK and FS will perform the statistical analyses. All authors provided comments, read and approved the final manuscript.

## Pre-publication history

The pre-publication history for this paper can be accessed here:


